# Application-Specific Optimization of Integrated Spectral Sensors

**DOI:** 10.1021/acsphotonics.5c01213

**Published:** 2025-07-23

**Authors:** D. M. J. van Elst, A. van Klinken, M. S. Cano-Velázquez, F. Ou, C. Li, K. D. Hakkel, M. Petruzzella, F. Pagliano, R. P. J. van Veldhoven, A. Fiore

**Affiliations:** † Department of Applied Physics and Science Education, Eindhoven Hendrik Casimir Institute, 3169 Eindhoven University of Technology, NL 5600 MB, Eindhoven, The Netherlands; ‡ 685177 MantiSpectra B.V., High Tech Campus 9, 5656 AE Eindhoven, The Netherlands

**Keywords:** spectral sensing, optical sensors, near-infrared, particle swarm optimization, integration, spectrometry

## Abstract

Near-infrared spectral sensing serves as a powerful technique for nondestructive analysis of material composition in a wide field of applications. A typical spectral sensor comprises an array of detectors, each with a response in a certain spectral band. We demonstrate an algorithm capable of tailoring these sensors for a specific near-infrared spectroscopy application by optimizing for all possible combinations of spectral bands. This approach outperforms manually selected designs, achieving high sensing performance even with just a few pixels. The results are confirmed by experiments on fabricated four-pixel devices, which feature a sensing accuracy exceeding the one of general-purpose sensors for a problem of practical relevance. This approach may enable cost-effective spectral sensors with simple read-out mechanisms for industrial and consumer applications.

## Graphical abstract

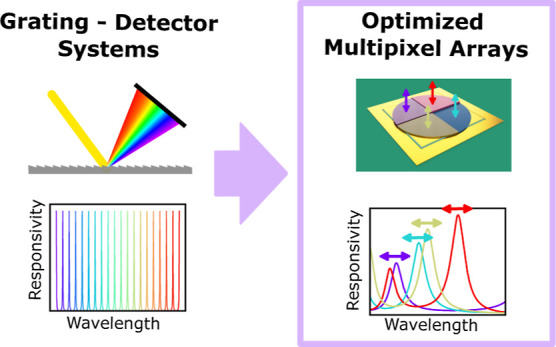

## Introduction

There is a growing interest in spectroscopy and spectral sensing for material analysis across a wide range of applications. Using light offers a fast and nondestructive approach to inspect the chemical composition of materials. The rapid advances in industry and consumer products have increased the demand for miniaturized, portable and cost-effective approaches to sensing.^1,2^

While laboratory equipment has superior resolution and spectral range, its high cost and bulkiness hinder its deployment in the field. Additionally, for most practical applications, ultrahigh resolution is not required for satisfactory performance. There are a variety of approaches to miniaturize the hardware for spectral measurements. The combination of gratings and detector arrays is a well-established approach for the measurement of the full spectrum with high resolution (see Figure 1a), but due to its reliance on free-space propagation for spectral dispersion it makes miniaturization and low-cost packaging challenging. Another approach is based on MEMS-tunable filters, which can result in integrated devices. However, due to their suspended structures they are susceptible to vibrations, and their speed is limited due to the need of scanning. As a final example, waveguide-based spectrometers are miniaturized to a great degree, but they are only suitable in combination with a single-mode input and therefore not suitable for measurements using inexpensive incoherent light sources.^3^

**Figure 1:**
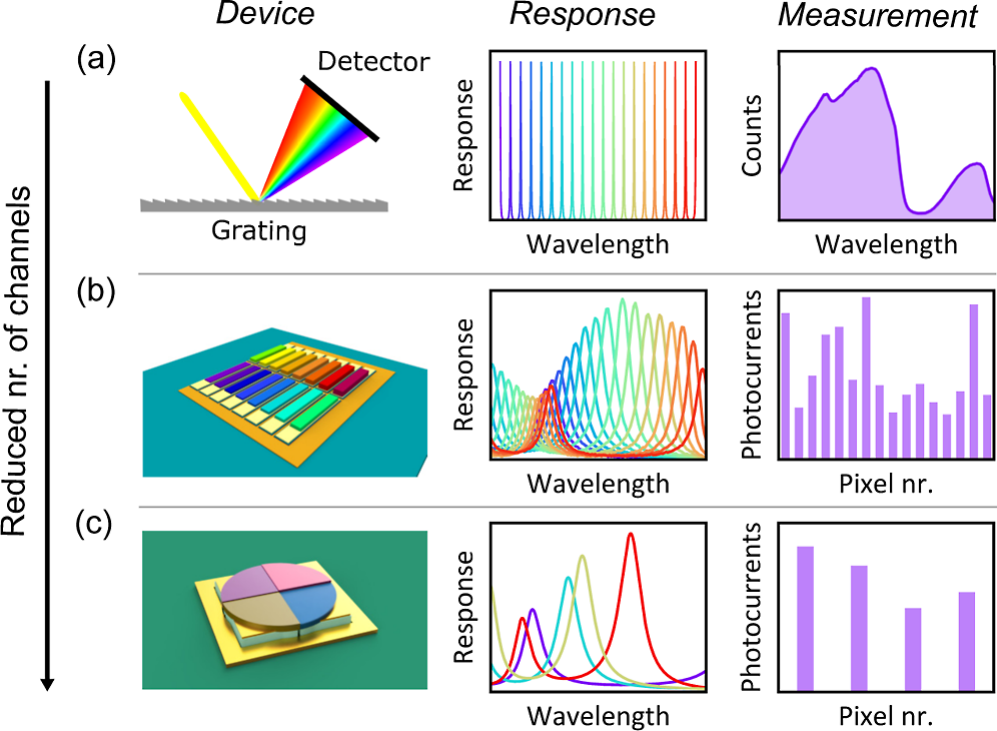
Simplifying the hardware for spectral sensing. (a) General grating-based spectrometers have high resolution with a large number of channels which allows a measurement of the full spectrum in the wavelength basis. (b) General-purpose spectral sensors, based on photodetector arrays integrated with filters, provide spectral information in the entire NIR range.^11,12^ (c) Compact spectral sensing arrays with fewer pixels achieve comparable sensing performance for lower hardware complexity due to their optimized responses.

As an alternative to high-resolution spectral measurements in the wavelength basis, a number of approaches have been proposed for integrated devices which rely on more complex filtering functions, often based on nanophotonic structures,^3–6^ including metasurfaces.^7–9^ These devices are commonly called “spectral sensors”, as they provide spectral information but not necessarily a spectrum–the spectrum can in some cases be “reconstructed” from the measured spectral data.^10^ However, most of these approaches rely on relatively complex fabrication schemes, are limited to single-mode input, and these complex filter structures often do not allow monolithic integration with the detectors.

Previously, a fully integrated spectral sensor designed for operation in the near-infrared (NIR) range (900–1700 nm) was demonstrated,^11^ fabricated using optical lithography.^12^ Similarly to visible-range spectral sensors,^13–15^ it does not measure the full spectrum but a limited number of spectral bands. To this aim it uses a 16-pixel array, where each pixel contains a thin absorbing layer and a tuning element within an optical cavity, resulting in a resonant-cavity enhanced (RCE) photodetector (see Figure 1b). The absence of movable or suspended components enhances the device’s robustness, making it resilient to vibrations encountered in on-field applications. As the most common measurement modality in NIR spectroscopy is diffuse reflectance, a relatively large device area of about ≈0.8 mm^2^ was chosen, ensuring efficient collection from a multimode fiber or from a spot of comparable size in free space. Furthermore, the scalability of the fabrication process makes it suitable for mass production. The effectiveness of this approach in practical applications was also demonstrated. In particular, it was demonstrated that spectral reconstruction is not needed, as accurate prediction models can be built directly using the sensor data.^16,17^

While this general-purpose NIR sensor can be deployed in a wide range of sensing problems, it is intuitive that for any specific problem a more optimized solution can be found by tailoring the spectral responses to the bands where most spectral information is available. The optimization of the spectral response of discrete filters (“Multivariate Optical Elements”, MOEs) was previously proposed,^18,19^ demonstrated for a number of sensing problems^20,21^ and recently commercialized.^22^ However, the complexity of the multilayer stacks needed to define the optimal filter responses makes the monolithic integration with the detectors, and the definition of arrays, very challenging.

In this work, we take a different and simpler approach to the optimization of a spectral sensor for a specific sensing problem: We choose the optimal spectral responses within a set available in an integrated filter-detector platform. Due to the smaller number of pixels needed (Figure 1c), the resulting spectral sensor has reduced fabrication and read-out complexity, while keeping the high level of integration and sensing accuracy of general-purpose NIR sensors.^11,12^ With this approach, these specialized sensors leverage a single technology platform and optimizing for a specific application only requires minor adjustments in the fabrication process. The approach is experimentally demonstrated by determining ethanol concentrations in aqueous solutions using a NIR spectral sensor with a few channels, but it can generally be applied to any sensing problem with known input spectra, using any sensor device for which the response can be modeled. The algorithm can find nontrivial solutions that outperform hand-picked designs, such as those based on latent variables (LV’s) or isosbestic points.^23^ The compact size and robustness of the resulting sensors pushes the field of portable sensing solutions^2,24,25^ and allows integration into existing devices such as smartphones^26^ and wearables for health monitoring,^27^ where resilience against vibrations is essential.

## Methods

### Application Case

As a case study for demonstrating the proposed approach, we choose the measurement of the ethanol concentration in aqueous solutions. Monitoring of this mixture is of importance to a variety of processes in the fuel and beverage industry.^28–31^ For the experiments, ten different mixtures of ethanol and distilled water were prepared with varying concentrations between 0% and 53%. The spectra of Figure 2b were measured in glass vials using a broadband light source (Ocean Optics HL2000), a transmission dip probe (Ocean Optics TP300-VIS-NIR, 300 μm fiber core diameter) and a fiber-coupled spectrometer (Avantes AvaSpec-Mini-NIR) (see Figure 2a). The mixtures were measured with a Krüss digital refractometer to obtain the refractive index at 589 nm, which is then converted to a concentration via the Gladstone–Dale relation^32^ and used as a calibration data for the optimization. To expand the data set and allow for a sufficient training/test split, 51 additional spectra are added by interpolating the signal at each wavelength against the concentration with a quadratic spline (see Figure 2b). However, the final experimental validation is always performed on experimentally measured spectra.

**Figure 2:**
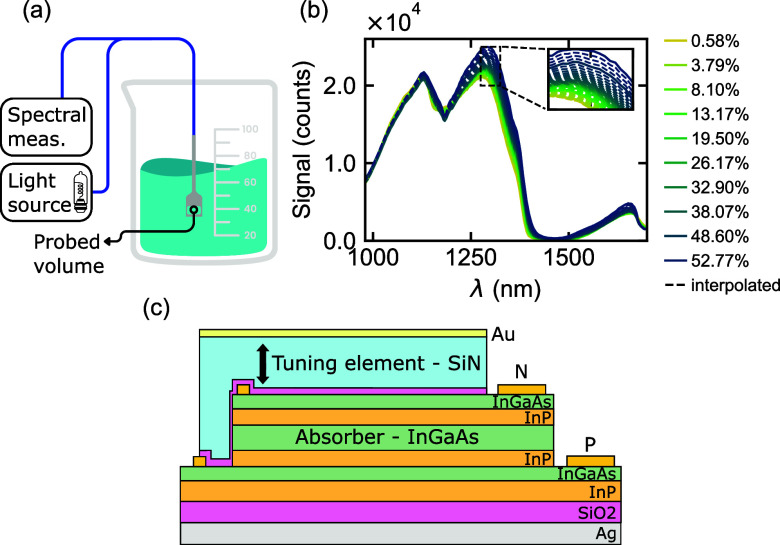
(a) Schematic of the experimental setup for transmission measurements in a liquid. (b) Measured transmitted spectra for varying concentrations of ethanol in water. The dashed lines represent interpolated data. (c) Schematic cross-section of the detector structure.

The ethanol/water solutions show distinctive features in the NIR range, and as shown below an accurate predictive model can be built based on the spectra. The question to investigate now is what prediction performance can be obtained using an integrated spectral sensor array with a limited number of pixels. The employed spectral sensor technology is based on previous work^12^ and consists of a p–i–n diode with an InGaAs absorber (Figure 2c). The diode is placed in an optical cavity along with a tuning element that specifies the resonant wavelength. We choose the mirror reflectance, thickness of the absorbing regions and the total cavity length in order to obtain 2–3 peaks with line width (FWHM) ≈50–60 nm and peak responsivities of R ≈ 0.15–0.35 A/W in the spectral window 900–1700 nm.^12^ High sensing performance with a 16-pixel array based on this device structure was previously demonstrated.^16,17^ The tuning layer consists of silicon nitride and its thickness is the parameter that is optimized in the algorithm for each pixel in the array. In order to make a fair comparison, when varying the amount of pixels used in different configurations of the sensor, the total sensing area is kept the same. This means that a 1-pixel device is expected to have 16 times the signal of a 16-pixel device. The responsivity curves required for the optimization can be calculated using the transfer-matrix method (TMM). This one-dimensional method for multilayer structures only makes use of the refractive index (RI) and thickness of each layer, allowing for fast computation.^33^

### Optimization Scheme

The application-specific optimization process is schematically represented in Figure 3. The input for the algorithm is a sensing problem characterized by known reflection or transmission spectra (for this case the spectra of Figure 2b) corresponding to a calibrated measurand *y* (ethanol concentration). In the optimization 60% of this data set is used and 40% is kept as test data for the final design. In order to predict the performance of a sensor device with arbitrary configuration for this problem, the responsivity curves have to be computationally simulated with reasonable accuracy, depending on the structure. A key aspect of our approach is that we fix the device structure, an InGaAs resonant-cavity detector,^12^ and optimize only the thickness of the tuning layer (see Figure 3c and detailed device description below). The photocurrents measured during a simulated experiment are calculated by integrating the incident spectra with the responsivity curves of each pixel, scaled with a fixed factor to obtain values in counts, representative of the read-out electronics. Gaussian noise with standard deviation σ, varying from 10^0^ to 10^3^ counts is applied to the photocurrents to simulate varying experimental conditions and ensure the convergence of the optimization. While the absolute values of photocurrent counts and noise counts are arbitrary in this simulation, we choose their ratio to match signal-to-noise (SNR) ratios of 10^2^ to 10^5^ typically observed in experiments with NIR spectral sensors (as shown below in the experimental results). We assume that the noise does not depend on the photocurrent or pixel area, which is representative of a common experimental situation where the readout electronics are the dominant noise source. As mentioned above, the signal scales with the number of pixels as ∝ *N*, given the assumption of constant area. The photocurrents are then used for Partial Least Squares (PLS) modeling,^34^ to obtain the average prediction strength of this configuration over all noise levels. For each design, the spectra are again split in training/test data (with a 60:40 ratio) and shuffled 25 times. For each training/test set, the PLS model is built using the training data and its prediction performance evaluated on the test data. The average Ratio of Performance to Deviation (RPD), which is the ratio of the standard deviation in the measurand (concentration) to the Root-Mean-Square Error (RMSE) of the prediction model, is used as the Figure of Merit (FOM). Using this FOM the design space is scanned using a particle swarm optimization (PSO), which is an algorithm that optimizes the target FOM by iteratively moving a population of candidate solutions (particles) toward the best solution based on their own and their neighboring particles’ positions and velocities.^35^ In this case the thicknesses of the tuning layers are optimized. A penalty is added to the FOM if the peaks are within 25 nm of each other to avoid clustering. Indeed, controlling the spectral separation between closely spaced peaks would be exceedingly difficult in practice. Here a PSO is preferred (over i.e. genetic optimization) due to the simplicity and physical understanding, reduced bookkeeping^36,37^ as well as its compatibility with parallel processing. Additionally, for larger number of pixels (8 or more), a combinatorial deposition approach is used to generate the tuning layer thicknesses with a reduced number of required lithography steps and thus parameters in the optimization.^38,39^ This means that a change in one parameter (the deposited thickness during one lithography step) effects the wavelength response of half of the pixels at the same time. This produces a complex parameter landscape with many local minima and hinders the use of straightforward gradient-descent methods. It is beneficial to have a large number of particles in the PSO,^40^ for this specific case with 5 parameters we use 1024 particles. Due to the large amount of solutions scanned per iteration this decreases the chance of missing a local optimum, enhances the robustness of the optimization and reduces the number of total iterations required for convergence to a solution. For the particle velocities the Matlab R2023a standards are used. We thus choose a PSO here due to its compatibility with our sensing device, but the choice of optimization method depends on the structure at hand and can be made more efficient by using for example hybrid methods.

**Figure 3:**
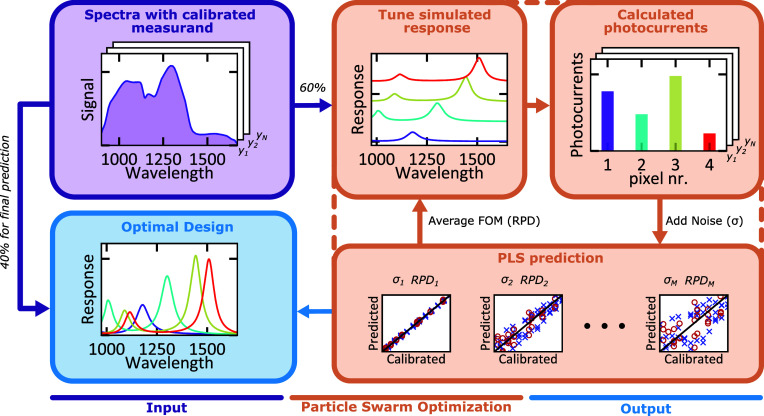
Schematic overview of the optimization algorithm. The input is a set of calibrated spectra. Then, the response of a spectral sensor configuration is tuned and its average performance evaluated over a range of noise levels. The dashed square indicates the process occurring within a particle swarm optimization. The result is a design with optimized performance for the given spectral sensing problem.

## Results and Discussion

### Results Optimization

The results of the optimization for different number pixels *N* can be seen in Figure 4a, providing the highest average RPD over the considered range of noise values σ = 10^0^–10^3^. They are then evaluated for single noise levels by keeping the design fixed and simulating the photocurrents with varying noise, as can be seen in Figure 4b. In this plot, σ = 1.3 corresponds to a SNR of 3.0 × 10^6^ for N = 1 (single detector) and a SNR = 2.8 × 10^4^ for N = 16. We note that for a given σ the SNR depends on the particular design. For all configurations of the sensor with 2 pixels or more, the prediction strength is good and can be seen to deteriorate with increasing noise as expected. Increasing *N* provides more spectral information, but also results in a lower SNR (due to the assumption of constant area), so it does not necessarily result in higher accuracy. This difference in SNR is the reason why 4-pixel and 8-pixel devices outperform 16-pixel devices in a broad range of noise values. As the *N* = 16 case closely corresponds to a general-purpose spectral sensor with pixels uniformly distributed over the spectrum, this clearly illustrates the added value of application-specific optimization in noise-sensitive cases: It results in simpler spectral sensors with less pixels, while also providing higher accuracy.

**Figure 4:**
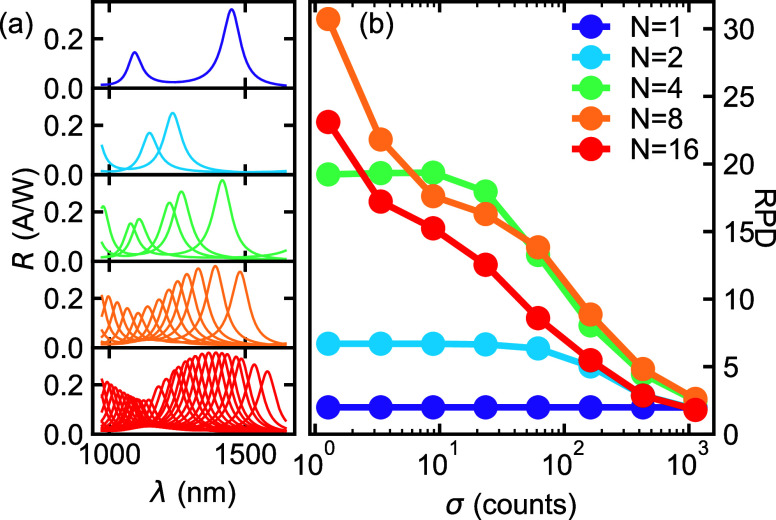
(a) Optimal designs as a result of the optimization for device structures with varying number of pixels (*N*). (b) Prediction accuracy of these designs for different noise levels.

As the most simple device with high sensing accuracy, the 4-pixel configuration was further investigated. Figure 5a shows the responsivity curves of the optimized design. For this sensor, the prediction result on the remaining test data (i.e., 40% of the data not used for the optimization) shows that when σ = 31 and SNR = 4.3 × 10^3^, an RPD = 18.6 is obtained (see Figure 5b) using a PLS analysis with 4 latent variables. This value is close to the simulated result observed in Figure 4b. Since the exact thicknesses of the SiN tuning layer can vary during the fabrication process, we further investigate the fabrication tolerance of this design. The robustness of this design against such fabrication deviations is shown in Figure 5c. A random variation Δ*t* on the tuning layer thicknesses *t*, according to a Gaussian distribution with a standard deviation σ_Δ*t*_, is applied to the SiN thickness. From there, the resulting responsivity curves are calculated and their prediction strength for the same noise level evaluated again. This is done 15 times for each point, and the corresponding average and standard deviation are shown as dots and error bars (as well as a shaded region), respectively, in Figure 5c. The prediction accuracy can be seen to decrease, but does not drop significantly, even for very large deviations of Δ*t* ≈ 15 nm. However, it can be seen that the variation of the prediction accuracy becomes larger, which is expected due to the increasing range of possible variations for larger deviations Δ*t*. In reality we expect an experimental control of the thickness corresponding to Δ*t* < 5 nm in a controlled production environment, which gives confidence in the fabrication feasibility of these structures.

**Figure 5:**
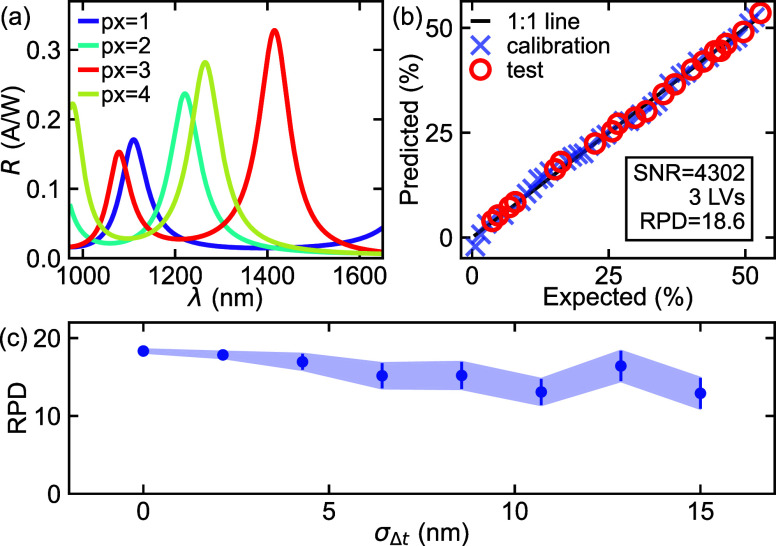
(a) Simulated responsivity curves of the 4-pixel sensor design. (b) Prediction accuracy of this array for σ = 31 noise, corresponding to a SNR = 4.3 × 10^3^ using 4 latent variables. (c) Robustness of the prediction strength against deviations in fabricated tuning layer thickness.

### Device Fabrication

The designed 4-pixel device of Figure 5a was fabricated with a circular structure (for optimized coupling to multimode fibers), following the process described in ref 12. The epistructure of the p–i–n diode (top to bottom in Figure 2) consists of 30 nm InGaAs (n-type, Si doping 1 × 10^19^ cm^–3^), 50 nm InP (n-type, Si doping 5 × 10^18^ cm^–3^), 100 nm InGaAs (absorber, nonintentionally doped), 50 nm InP (p-type, Zn doping 1 × 10^18^ cm^–3^), 30 nm InGaAs (p-type, Zn doping 1 × 10^19^ cm^–3^), 80 nm InP (p-type, Zn doping 1 × 10^18^ cm^–3^). A microscope image of the device used for further measurements can be seen in Figure 6a, where the different colors are due to the varying thicknesses of the tuning layer. The thicknesses of the SiN tuning layers (deposited by inductively coupled plasma-enhanced chemical vapor deposition) were determined with a Filmetrics reflectometer on separate silicon test pieces in the same chamber and were within Δ*t* < 2 nm of the design (root-mean-square error = 1.2 nm). The responsivity of each pixel is determined by measuring the photocurrent under monochromatic illumination. The monochromator (Spectral Products Digikröm CM110) uses a broadband-light source and 850 nm long-pass filter at the input. The output is coupled to a multimode fiber (core diameter of 550 μm) and features a spectral line with FWHM ≈8 nm. The light is focused onto the optically active area of the pixel using a microscope. Figure 6b shows the responsivity curves measured on one of the devices with the closest spectral match to the design, along with the simulated spectra. A blue-shift is observed on all peaks, with an average wavelength deviation $\overset{®}{\left|\Delta \lambda \right|}$ = 17.2 nm. Changes from the design in wavelength and amplitude, aside from differently deposited thicknesses, are attributed to thickness variations in the epitaxially grown diode layers and changed state of the plasma deposition tools resulting in different optical properties and inhomogeneous thicknesses of deposited dielectric layers throughout the chamber. These deviations are not unexpected in research growth and fabrication equipment. The spread in the resonant wavelengths of each mode due to these variations is approximately Δλ ≈ 12–40 nm, as observed across other fabricated arrays.

**Figure 6:**
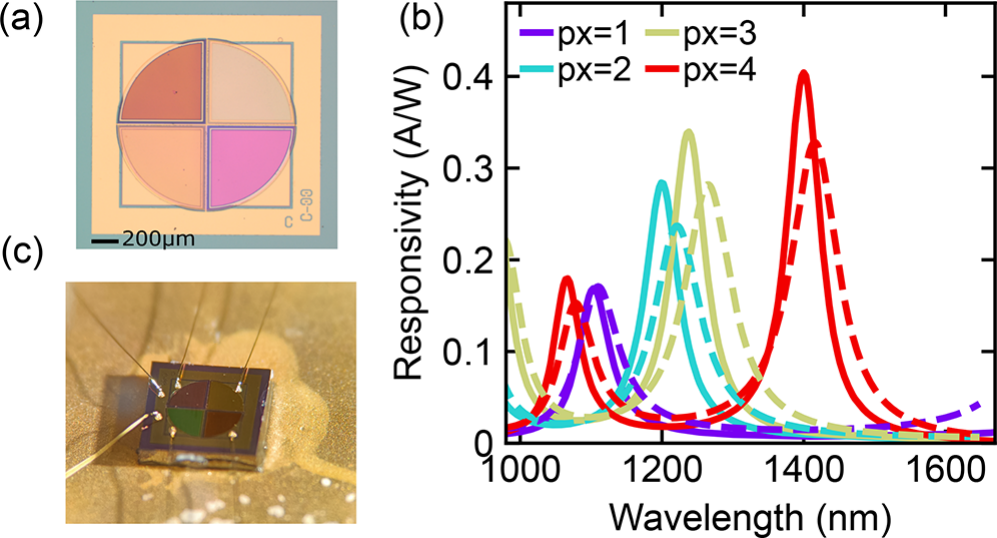
(a) Microscope image of the fabricated 4-pixel sensor used in the experiments. (b) Experimentally measured responsivity curves (solid lines) and the optimized design (dashed lines). (c) 4-pixel array mounted and wirebonded on a chip carrier.

The array of Figure 6 was then mounted on a chip carrier and wirebonded for read-out via an electronic board (Figure 6c). The pixels are sequentially read out with a total read-out time of approximately 1.1 s, because the electronic board was designed for a 16-pixel system. First, signals from the sensor array and the remaining 12 unused inputs are amplified, followed by multiplexing and digitization using a 24 bit analog-to-digital converter (ADC). Finally, a microprocessor interrogates the ADC and relays the data to a computer via a USB connection.^16^

### Validation of Fabricated Optimized Device

For the validation experiments the spectral signal from the transmission probe (see in Figure 3a) was split into two signals using a Y-splitter (Thorlabs 400 μm core, 0.39NA, 50:50 coupler). One fiber was used to measure the spectra for reference, whereas the other was focused onto the wirebonded chip with a simple 2-lens setup (Plano-Convex f = 25 mm and f = 75 mm) and an 850 nm long-pass filter. For this experiment new mixtures of ethanol in distilled water between 0% and 54% were made and calibrated with a refractometer to obtain the refractive index and calculate the corresponding concentration.

The resulting measured spectra and photocurrents can be seen in Figure 7a,c. Both are sum-normalized before chemometric analysis, as this processing method makes it robust against intensity variations from the light source. For the full spectra, PLS with 4 LV’s and 5-fold cross-validation was used resulting in an average RPD = 31.0 ± 3.7, with no notable discrepancies between the splits. This corresponds to a RMSE = (0.59 ± 0.07)% (see Figure 7b). The same processing is applied to the data from the 4-pixel array, giving an RPD = 21.3 ± 3.1 with RMSE = (0.87 ± 0.12)%, indicative of a strong model (see Figure 7d). The SNR of the 4-pixel array in this experiment was SNR ≈ (2.6–3) · 10^4^, determined by taking the standard deviation over 100 repeated measurements in distilled water (i.e., 0% ethanol). In the simulated performance of Figure 4b this would correspond to σ ≈ 15 and a RPD ≈18, showing a good agreement between the simulation results and the experimental performance. The accuracy obtained is not as good as the one obtained using other NIR methods, which utilize large benchtop instruments, which is on the order of 0.1–0.2% RMSE of prediction (volumetric, v/v %).^41^ However, it is a remarkable performance considering the compact size and affordability of our sensor, which would allow its use within processing equipment. This performance can be further improved by extending the size of the training data set, as well as by improving the fabrication tolerance to ensure a better match to the design, which is readily achieved in an industrial microfabrication process.

**Figure 7:**
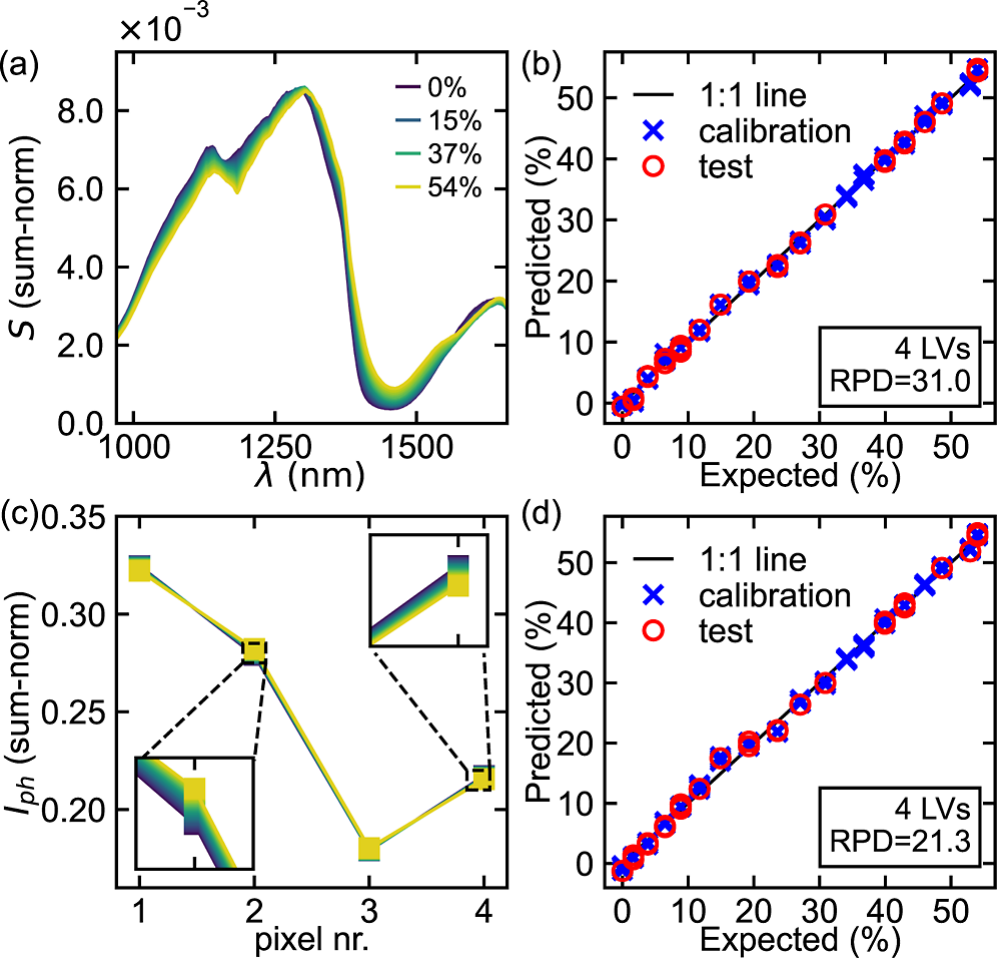
Results of the validation experiment on the optimized 4-pixel device. (a,b) Sum-normalized spectra measured with a commercial spectrometer for varying concentrations of ethanol in water and a PLS prediction using 4 latent variables of this concentration. (c,d) Sum-normalized measured photocurrents with the optimized array during the same experiment as panel (a) and a PLS prediction using 4 latent variables of the concentration.

We note that the overall sensing performance in a given application setting could be affected by potential interferents (e.g., other chemicals). While an evaluation of these effects goes beyond the scope of this work, we note that the presence of interferents can be taken into account in the optimization algorithms to provide the most robust design. There are a variety of ways to incorporate the effects of such interferents in the optimization, we give two possible strategies here. Ideally, one would acquire experimental data with heavily polluted samples, either with known or unknown concentrations of the contaminant. This will reduce the accuracy of the quantity to be determined, but increases its robustness to the interferent. If no experimental data is available, the effect of interferents can be modeled as random perturbations to the generated spectra. Similar to the modeled noise on the read-out photocurrents, this will mask the signal and generate a preference for designs that are most tolerant to interferents.

### Comparison to General-Purpose Spectral Sensors

For comparison we also repeated the same experiment with a general-purpose, not-optimized 14-pixel spectral sensor^12^ (see Supporting Information Supplementary Section 1). The similarity in the experimental conditions was verified (by achieving comparable prediction performance with the spectrometer used in both experiments) and the resulting PLS of the prediction gave an RPD = 12.9 ± 1.9, which is a strong model, but with significantly lower accuracy than the one of our optimized 4-pixel sensor. This is generally in line with the trend observed in Figure 4b and provides additional strong evidence of the gain provided by the optimization. We stress that this gain is obtained under realistic, noisy experimental conditions and despite the nonideal matching between the responsivity spectra of the designed and fabricated devices, showing the potential of our method in a practical setting. Further optimizing the read-out specifically for the reduced number of pixels allows an additional gain in SNR for a given integration time.

### Optimal Response of Generic Spectral Sensors

The above demonstrated results showed the gain in performance that can be obtained for this specific case study and sensing device. However, the approach is applicable to any sensing problem with known spectra and for which the response can be modeled. Thus, it is also possible to evaluate the optimal configuration of a generic spectral sensor consisting of an array of pixels with different spectral response in general, without the constraints of this resonant-cavity detector structure. A demonstration of this is shown in Supporting Information Supplementary Section 2. Here, sensor configurations consisting of 1 to 256 channels with FWHM varying from 1 to 101 nm are evaluated for three common NIR sensing problems (ethanol concentration, milk fat and rice moisture). The sensor responsivity spectra are assumed to be simple Lorentzians, and a constant total area is assumed, implying that the signal in one channel scales inversely with the number of channels. The simulated results point to regions of optimal performance, which tend to have a moderate amount of channels (4–8) with broad line widths. This is in line with our findings for the RCE structure and indicates that they generally apply to spatially multiplexed spectral sensor arrays. Adding channels on the same sensing area reduces the signal throughput as ∝ 1/*N* and reducing the line widths of the detectors further decreases the measured signal, which reduces the prediction accuracy. This was also pointed out in the analysis by Haibach and Myrick.^42^ The value of optimizing a read-out system was also demonstrated in recent works from our group,^43,44^ which further illustrates the advantages of sensors with a reduced number of channels. It should be noted that the applicability of these findings depends on the sensing problem at hand and particularly on the width of the spectral features.

## Conclusions

In conclusion, our work introduces a versatile approach for optimizing spectral sensors to a specific application. By leveraging just a few pixels, high sensing performance can be achieved. Optimizing for all combinations of spectral bands simultaneously yields nontrivial design choices. Notably, in noisy environments fewer pixels within the same sensing area can outperform a higher number of channels due to an improved signal-to-noise ratio (SNR). We have provided an experimental demonstration of this approach and fabricated an optimized 4-pixel NIR spectral sensor which shows high prediction performance in the measurement of ethanol/water solutions, and outperforms a general-purpose spectral sensor. This technique can be adapted to any sensing problem that has known input spectra, assuming the spectral sensor pixels can be fabricated with reasonable accuracy.

## Supplementary Material



## Data Availability

The data can be found in an open access repository on Zenodo (van Elst, D. M. J. (2025). Application-Specific Optimization of Integrated Spectral Sensors. ACS Photonics, DOI:10.5281/zenodo.12083675).

## Notes

Supporting section S1 and S2 (PDF)

## §

College of Information Science and Electronic Engineering, Zhejiang University, 310027 Hangzhou, China and Research Center for Intelligent Optoelectronic Computing, Zhejiang Lab, 311121 Hangzhou, China
