# Supplementary material for: Application-Specific Optimization of Integrated Spectral Sensors
Source: ACS Photonics. 2025 Jul 23;12(8):4723–30. doi: 10.1021/acsphotonics.5c01213 (PMC12372167; doi:10.1021/acsphotonics.5c01213)
Supplement: Supplementary file 1 [file ph5c01213_si_002.pdf]

Supplementary Information: Application-Specific Optimization of Integrated Spectral Sensors

D.M.J. van Elst,*,† A. van Klinken,† M.S. Cano-Velázquez,† F. Ou,†,‡ C. Li,†,¶ K.D. Hakkel,†,‡ M. Petruzzella,†,‡ F. Pagliano,† R.P.J. van Veldhoven,† and A. Fiore†

†*Department of Applied Physics and Science Education, Eindhoven Hendrik Casimir Institute, Eindhoven University of Technology, PO Box 513, NL 5600 MB Eindhoven, The Netherlands.*

‡*MantiSpectra B.V., High Tech Campus 9, 5656 AE Eindhoven, The Netherlands.*

¶*currently employed at College of Information Science and Electronic Engineering, Zhejiang University, 310027 Hangzhou, China and Research Center for Intelligent Optoelectronic Computing, Zhejiang Lab, 311121 Hangzhou, China.*

E-mail: d.m.j.v.elst@tue.nl

Comparison to previous work

To compare the performance of the 4-pixel array to previous work on general-purpose NIR spectral sensors,[1] the experiment was repeated in the exact same way using a chip with 14 active pixels of a 16-pixel device (see Figure S1a, fabricated as described in[1]). Note that this array was characterized directly on the chip, thus only allowing a measurement in counts. The signal has been normalized to the power incident on the chip through the

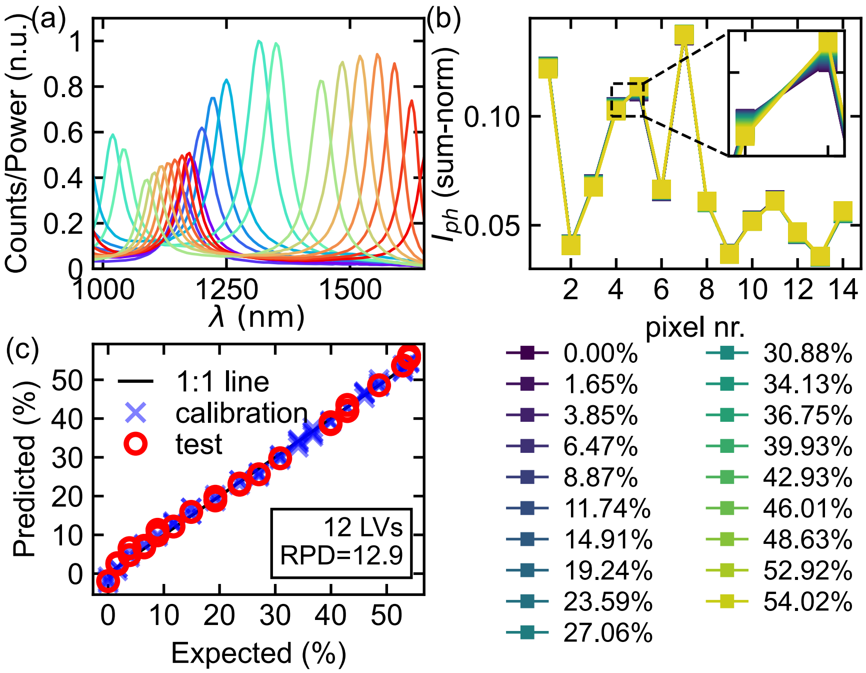

Figure S1: Results of a reference experiment using a chip as in[1] with 14 active pixels. a) Response curves of the array used in the experiment. b) Sum-normalized photocurrents of measurements in solutions with varying concentrations of ethanol in water. c) PLS prediction to determine this concentration.

free-space optical setup used for characterization for relative comparison between the pixels. The peak responsivity values here correspond roughly to ≈0.3 A/W as seen in comparable arrays.[1] In order to confirm the similarity in the experimental conditions, the full spectra were also measured in this experiment and the corresponding PLS model gave a 5 times cross-validated RPD $= 30.2 \pm 4.7$, showing a high level of reproducibility of the experiment. The measured photocurrents and the corresponding prediction can be seen in Figure S1b,c. PLS with 12 latent variables and 5-fold cross-validation was used resulting in an average RPD $= 12.9 \pm 1.9$, with no notable discrepancies between the splits. This corresponds to an RMSE $= (1.44 \pm 0.27)\%$. These values are significantly worse than the ones obtained with the optimized 4-pixel device. The SNR of the 14 pixel array in this experiment was SNR $\approx (1.1\text{-}1.4) \cdot 10^4$, which is about a factor of two lower than the 4-pixel array. The ratio of the active pixel areas (a factor of 6) between the two devices is even larger than the ratio of SNRs, but this difference could be due to other factors contributing to the SNRs, such as

the optical coupling to the array and the noise in the detectors.

Optimal configuration of a generic spectral sensor based on spatial multiplexing

Approach

In the main text the constraints on the sensor design only allowed optimizing the positions of the resonant features, while the linewidths were fixed by the resulting structure. In this section we investigate what the optimal configuration of a custom-build spatially-multiplexed spectral sensor array is for general NIRS problems without such constraints. This spectral sensor has N channels with a certain linewidth FWHM. The responsivities are assumed to be single Lorentzians, with a fixed peak responsivity of $R = 0.4$A/W and varying positions. Similar to the main text, the sensing *area* of is kept constant, so adding more channels means each channel loses SNR proportional to SNR $\propto 1/N$ (this assumes the dominant noise contribution comes from the electronic readout and is independent of the area). For other spectral hardware this assumption can be adapted. Additionally, the photocurrents are calculated as an integral of the incoming spectra and the response curves, meaning that narrow linewidths will result in lower signals. The sets of responsivity curves are evaluated for 4 different levels of white noise with a Gaussian distribution of width $\sigma = 10^1 - 10^2$ (corresponding to the middle range of the simulations of the main text) and the average RPD is the FOM. The corresponding SNR is displayed in the figures as well. In order to scan a wide range of channels and linewidths, the optimization is simplified to 3 iterations with 1024 particles. This means that a total of 3072 possible configurations are scanned, giving a satisfactory crude optimized configuration. Only for sensors with 32 or less channels the positions are optimized. For 64 channels or more the configuration is equally spaced throughout the range, as it would already fully cover the range for linewidths of 10 nm or

larger. Also, PSO is not an optimal approach for particles with so many degrees of freedom. In the following sections this optimization is performed for three common NIRS datasets: ethanol concentration in water, fat content in raw cow milk and the moisture content in rice. The photocurrent values of these three different cases are scaled to achieve comparable SNRs, as expected in an experimental setting.

Ethanol sensing

As a first case study, the same input spectra as the main text are used for varying ethanol concentrations in aqueous solutions (see Figure 2b). Figure S2a shows the achieved prediction strength with varying combinations of linewidth and number of channels. The corresponding SNRs of these sensor configurations are given in Figure S2b. The bottom row gives examples of optimized configurations for the fixed linewidth FWHM = 11nm. In Figure S2a the top 20% highest RPD sensors are indicated by the dashed line. These lines mostly enclose a region with a moderate number of channels, namely 4 or 8, and linewidths ranging from 40 to 100 nm.

Milk fat sensing

The same investigation is applied to another general NIR spectroscopy case of sensing fat content in raw cow milk,[1,2] with spectra taken from Ref.[3] The results are shown in Figure S3. Again, the top 20% highest performing solutions encompass a region of relatively low number of channels with large linewidths.

Rice moisture sensing

Finally, another interesting case of sensing the moisture content in rice, for which we use spectra measured in Ref.[4] Again, a similar region of optimal performance is observed.

The analysis of these three general NIRS cases indicate that sensors with a moderate number

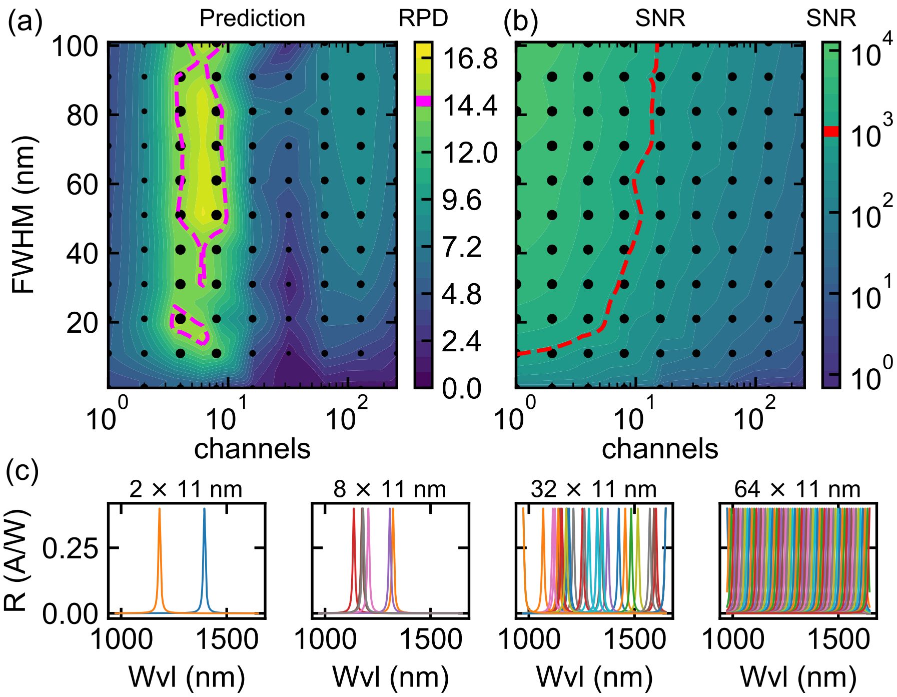

Figure S2: Optimal sensor configuration for the determination of ethanol concentration in water. a) Prediction strength of spectral sensors with N number of channels with a Lorentzian response of varying linewidths at fixed Responsivity (0.4 A/W). The configurations with 32 or less pixels have optimized positions. b) SNR of the same designs. The black dots indicate the simulated designs. c) Examples of sensor configurations with 11 nm linewidth and varying number of channels.

of channels and a linewidths of a few tens of nm are preferred over nm-range resolutions. The representative nature of the above sensing cases indicates the broad applicability of efficient, compact devices with optimized responses.

References

(1) van Klinken, A.; van Elst, D. M. J.; Li, C.; Petruzzella, M.; Hakkel, K. D.; Ou, F.; Pagliano, F.; van Veldhoven, R.; Fiore, A. High-performance photodetector arrays for near-infrared spectral sensing. *APL Photonics* **2023**, *8*, 041302.

(2) Hakkel, K. D.; Petruzzella, M.; Ou, F.; Liu, T.; Pagliano, F.; Veldhoven, R. P. J. V.;

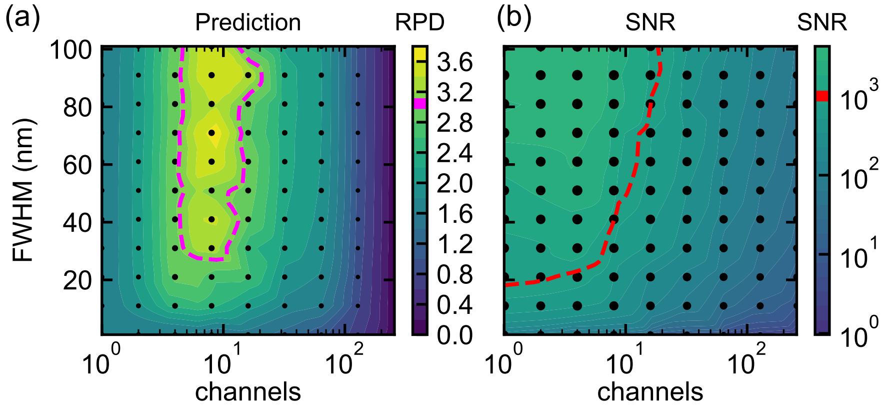

Figure S3: Optimal sensor configuration for the determination of the fat content in raw cow milk. a) Prediction strength of spectral sensors with N number of channels with a Lorentzian response of varying linewidths at fixed Responsivity (0.4 A/W). The configurations with 32 or less pixels have optimized positions. b) SNR of the same designs. The black dots indicate the simulated designs.

Fiore, A. Integrated near-infrared spectral sensing. *Nature Communications* **2022**, *13*, 1–8.

(3) Aernouts, B.; Polshin, E.; Lammertyn, J.; Saeys, W. Visible and near-infrared spectroscopic analysis of raw milk for cow health monitoring: reflectance or transmittance? *Journal of dairy science* **2011**, *94*, 5315–5329.

(4) Ou, F.; van Klinken, A.; Ševo, P.; Petruzzella, M.; Li, C.; van Elst, D. M.; Hakkel, K. D.; Pagliano, F.; van Veldhoven, R. P.; Fiore, A. Handheld NIR Spectral Sensor Module Based on a Fully-Integrated Detector Array. *Sensors* **2022**, *22*, 7027.

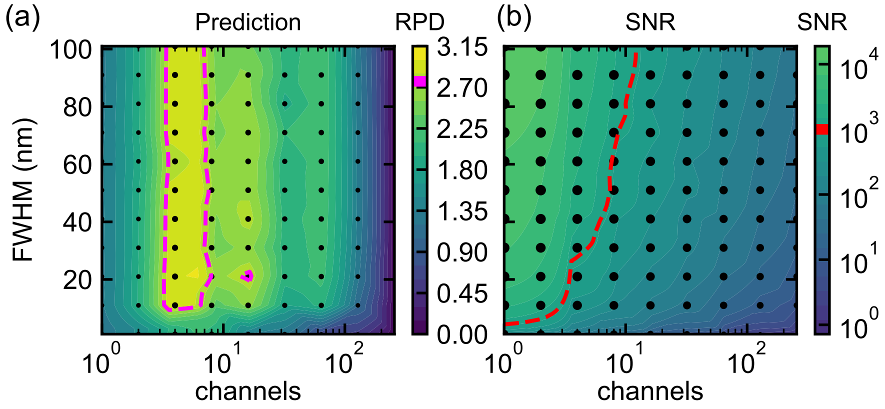

Figure S4: Optimal sensor configuration for the determination of rice moisture concentration. a) Prediction strength of spectral sensors with N number of channels with a Lorentzian response of varying linewidths at fixed Responsivity (0.4 A/W). The configurations with 32 or less pixels have optimized positions. b) SNR of the same designs. The black dots indicate the simulated designs.
